# Disinfection of incubators in neonatal intensive care units: impact of steam pulverization on bacterial colonization

**DOI:** 10.1186/s13756-023-01226-y

**Published:** 2023-03-16

**Authors:** Marion Reboux, Marie Chavignon, Anne Tristan, Franck Plaisant, Frédéric Laurent, Marine Butin

**Affiliations:** 1 Service de Néonatologie et Réanimation Néonatale, Hôpital Femme Mère Enfant, Hospices Civils de Lyon, 59 boulevard Pinel, 69500 Bron, France; 2 Team « Staphylococcal Pathogenesis », Faculté de médecine Laennec, Centre International de Recherche en Infectiologie, INSERM U1111, 7 Rue Guillaume Paradin, 69008 Lyon, France; 3 Institut des Agents Infectieux, Centre National de Référence des Staphylocoques, Hospices Civils de Lyon, 103 Grande Rue de la Croix Rousse, 69317 Lyon Cedex 04, France

**Keywords:** Neonatal intensive care units, Neonatal incubators, Bacterial contamination, Disinfection, Steam pulverization

## Abstract

**Background:**

In neonatal intensive care units (NICUs), neonates requiring medical care after birth, including very vulnerable preterm infants, are housed in incubators. Previous studies have reported that the standard chemical disinfection measures used to disinfect these incubators are insufficient to eradicate contaminating bacteria, leading to a worrying infectious risk for preterm neonates. This study aimed to evaluate the efficacy of a disinfection method based on steam pulverization to eradicate the persistent bacterial contamination in such incubators.

**Methods:**

In a tertiary NICU, 20 incubators were monitored qualitatively for bacterial contamination at five different sites (the rubber grommet, the left door handles, the temperature adjustment button, the mattress and the scale) using a culture method at three times: before and after steam pulverization then 24 h after turning on and housing a new neonate. Clinical data of neonates housed in each incubator were retrieved from the medical records to identify potential occurrence of late onset sepsis (LOS).

**Results:**

Just after steam pulverization, only two incubators were free from bacteria. Before disinfection 87% of all the samples were contaminated compared to 61% after disinfection. After 24 h, the proportion of contaminated samples reached 85%. Mattresses and scales were the most frequently contaminated incubator sites with respectively 90% and 80% positive samples after disinfection compared to 100% and 90% before disinfection. Coagulase-negative staphylococci, *Enterococcus, Enterobacteria* and *Bacillus* resisted disinfection and were identified on respectively 90%, 20%, 5% and 45% of incubators just after disinfection. Three preterm neonates developed LOS after being housed in a disinfected incubator but the bacterial species involved have not been identified in their incubator after disinfection. In two cases, the bacterium had been isolated from the mattress 24 h after housing the infected patient.

**Conclusion:**

Steam pulverization is not sufficient to eradicate bacterial contamination of incubators. These results highlight the urgent need for an effective disinfection method, especially for mattresses that are in constant contact with patients. In parallel, new incubator designs and mattress protections must be developed.

## Background

In neonatal intensive care units (NICUs), late onset sepsis (LOS) are frequent and responsible for increased morbidity and mortality, especially among very low birth weight preterm infants [1]. According to the literature, LOS are of nosocomial origin in 78–80% of the cases and involve multidrug resistant bacteria including coagulase-negative staphylococci (CoNS), *Staphylococcus aureus* and *Enterobacteriaceae* [1–4]. Among CoNS, a special attention is given to the pathogen *Staphylococcus capitis* NRCS-A that has been shown to be specifically involved in LOS in NICUs worldwide [5]. In NICUs, preterm neonates are in close contact with their environment including their hosting incubator and the medical equipment but also with their parents and caregivers, which are all potential bacterial reservoirs. If hygiene measures, especially hand washing, substantially decreases the risk of pathogen transmission from caregivers/parents to hospitalized neonates, the environment remains a major reservoir of pathogenic bacteria despite frequent disinfection procedures [6–8]. In particular, a previous study has shown that the origin and reservoir of *S. capitis* NRCS-A are not mothers nor caregivers, but the environment [9]. Data from the literature, as well as previous data obtained by our research team, suggest that numerous pathogenic bacteria are able to persist in the environment notably on incubators [9–12]. A careful and effective disinfection of incubators is thus pivotal to limit contamination and inter-patients transmission of pathogenic strains in NICUs [10].

In that regard, in a previous study we highlighted that the standard disinfection method based on the use of a detergent/disinfectant bath in our tertiary NICU was not able to eradicate pathogens from incubators, especially CoNS including *S. capitis* NRCS-A [12]. Thus, all incubators remained contaminated with at least one potential pathogen just after disinfection. This was consistent with data from other authors showing that incubators can act as reservoirs of pathogens involved in outbreaks in NICUs [9, 13, 14]. Difficulties in incubator disinfection could be related to their design and the materials used for their manufacture since many delicate parts and recesses cannot be immersed in detergent/disinfectant, especially the electronic scale and the mattress that have been shown to constitute the mostly contaminated parts of incubators [9, 12]. In addition, bacterial characteristics including biofilm synthesis or tolerance to detergent/disinfectants may explain the difficulties of eradication from abiotic surfaces and pave the way for the development of other disinfection approaches [7, 15, 16]. Alternatives to chemical disinfection have been suggested by other authors and have been tested in the hospital environment. They include UV irradiation which damages microbial DNA at specific wavelengths, high-intensity narrow-spectrum light irradiation that induces production of highly reactive bactericidal compounds, ozone which is an oxidizing agent highly effective against vegetative bacteria but toxic and corrosive, and steam technology [17]. The steam technology, combining high temperature (130–150 °C) and pressure (4–6 bars), fosters access to recesses impossible to reach using chemical methods, without producing toxic residues or creating bacterial selection pressure [18]. This disinfection approach is thus safe for neonates and caregivers and could represent a very suitable disinfection method in NICU settings [19, 20].

The aim of the present study was to evaluate the effectiveness of the newly implemented steam pulverization protocol for incubator disinfection in a tertiary NICU in real life conditions and to compare it with the results previously obtained with the standard chemical method. The secondary objectives were to describe the early re-contamination of incubators after disinfection. Finally, a follow-up of the patients housed in each incubator was performed for the development of LOS.

## Methods

### Study setting and period

This non interventional study aimed to evaluate the effectiveness of a disinfection protocol based on steam pulverization for incubator disinfection in the NICU environment. It was conducted in a 50-beds tertiary NICU (Hôpital Femme-Mère-Enfant, Hospices Civils de Lyon, France), the same place where a previous study evaluating the efficacy of the standard chemical disinfection protocol had been conducted [12]. The protocol of steam pulverization for incubator disinfection was implemented in this NICU in June 2021. A delay of six months between the change in the protocol of disinfection and the start of the present study was introduced to ensure a sufficient expertise of the caregivers in performing this new protocol. The study was conducted during three consecutive weeks in December 2021 during which the efficacy of steam disinfection was prospectively evaluated.

### Procedure of incubator disinfection

All incubators requiring disinfection during the study period were consecutively included in the study until a total of 20 incubators. Two types of incubators were used in the NICU during the study: Giraffe TM (General Electrics Healthcare, Limonest, France) and SATIS+ (Médipréma, Tauxigny, France). All incubators were changed after 7 to 10 days and disinfected in a dedicated room by qualified caregivers. Standard hygiene precautions were applied including the wearing of an impermeable gown and gloves during the dismantling of the incubator and its disinfection. Hydro alcoholic solution was used for hand disinfection before disinfection and between each step.

Steam pulverization was carried out using a steam generator (SP400, Sanivap, Sainte-Concorce, France) according to the manufacturer recommendations. All removable parts were cleaned and disinfected after dismantling of the incubator. The steam was pulverized at most three centimeters from the surfaces by an angled handle allowing access to difficult areas. The disinfection was carried out from the upper to the lower parts of the incubator. After disinfection, all incubator parts were dried with a microfiber wipe before the reassembly of the incubator.

### Bacterial culture and identification

The surface of 5 parts of each incubator (the rubber grommet, the left door handles, the temperature adjustment button, the mattress and the scale) was sampled once just before disinfection and just after disinfection using flocked swabs (ESwab, Copan, United States). These sites were chosen because they were the most frequently touched and colonized in previous studies [9, 12, 21]. Two sites (the rubber grommet and the mattress) were also swabbed once after 24 h of housing a new neonate. Thus, a total of 100 samples (corresponding to 5 sites for each of the 20 included incubators) were obtained both before disinfection and after disinfection and 40 samples after 24 h of housing a new neonate.

Contamination of a surface was defined by the presence in culture of at least one bacterial strain belonging to a species classically involved in neonatal LOS able to growth on selected media i.e. CoNS (including *S. capitis* NRCS-A), *S. aureus*, *Enterobacteriaceae*, *Enterococcus*, and *Bacillus* species [12]. For each sample, after 24 h of incubation in brain heart infusion (BHI-T, BioMérieux, Marcy l’Etoile, France) at 37 °C, two agar plates were inoculated with the suspension: one MRSA Brillance 2 (Oxoid®, Versel, Germany) to isolate methicillin-resistant staphylococci including *S. capitis* NRCS-A as previously described [22] and one CPSE agar plate (Chrom ID® CPS® Elite, Biomérieux, Marcy l’Etoile, France) to isolate other bacteria. Species identification was performed by matrix-assisted laser desorption-ionization-time of flight mass spectrometry (MALDI-TOF MS) using the VITEK® MS system (BioMérieux, Marcy l’Etoile, France).

### Collection of clinical data

Following incubator sampling and identification of the bacterial contaminants, clinical data including demographical data and occurrence of LOS were retrospectively collected from the electronic medical records (ICCA software, Philips®, Suresnes, France) for each neonate housed in one of the twenty incubators cleaned by steam pulverization during the study period. LOS was defined by (i) suggestive clinical or biological disorders in a patient older than three days, associated with at least one positive culture of either blood, tracheobronchial suction or cerebrospinal fluid, in accordance with the criteria usually used in the literature [12, 23] and (ii) confirmation of the diagnosis by the physician in charge of the patient at the time of infection and/or prescription of an antibiotic treatment for at least five days.

## Results

### Efficacy of incubator disinfection

During the study, all the 20 incubators were included in the analysis. Based on the protocol used, all 20 incubators were contaminated before disinfection and only two (10%) showed a total bacterial eradication in any sites after disinfection.

**Fig. 1:**
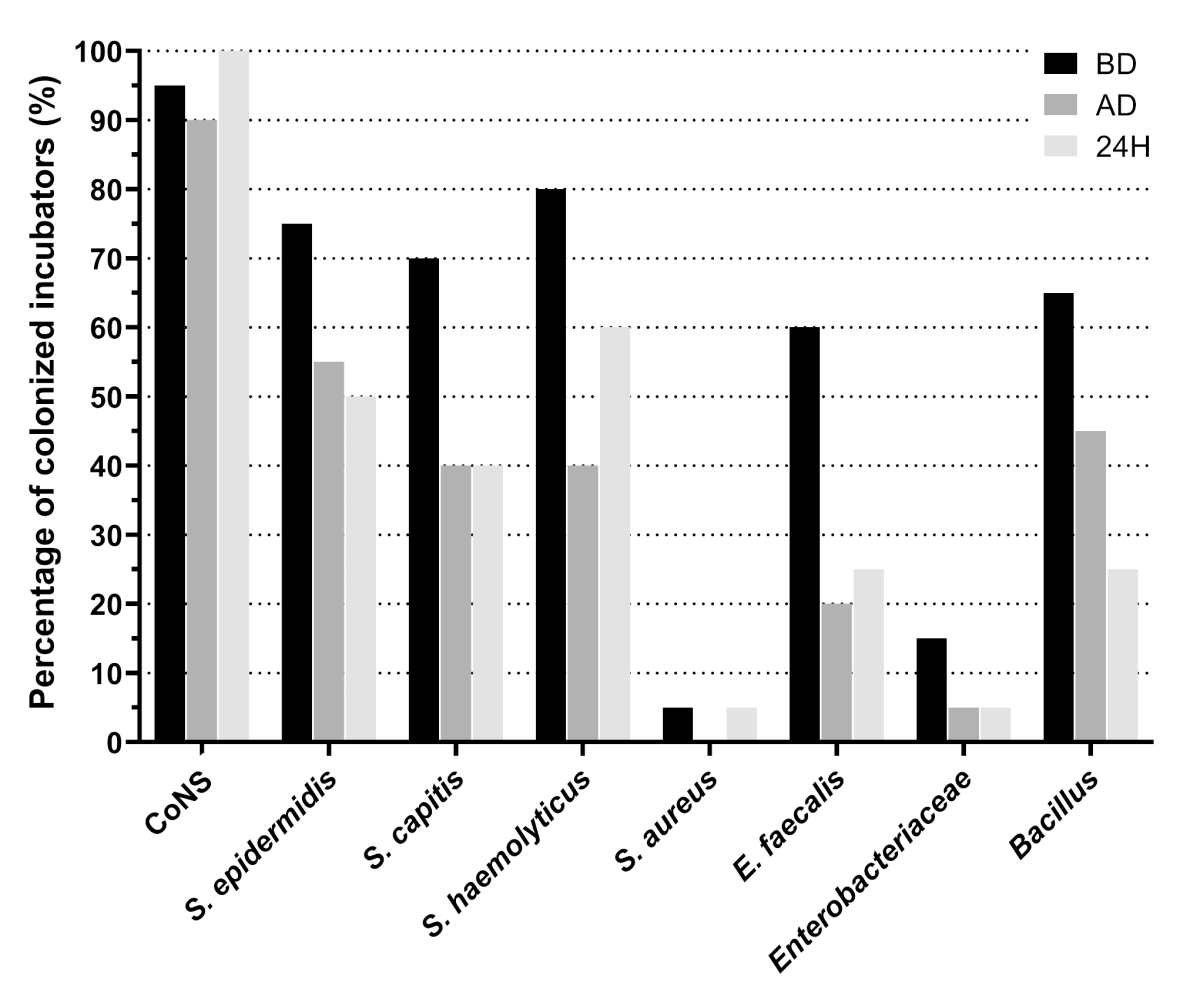
Bacterial species isolated from the 20 incubators. Data are presented as percentage of incubators that were colonized with each bacterial species/group/family among the 20 incubators of the study at the different times of the study: before disinfection (BD), after disinfection (AD) and after 24 h of housing a neonate (H24)

**Fig. 2:**
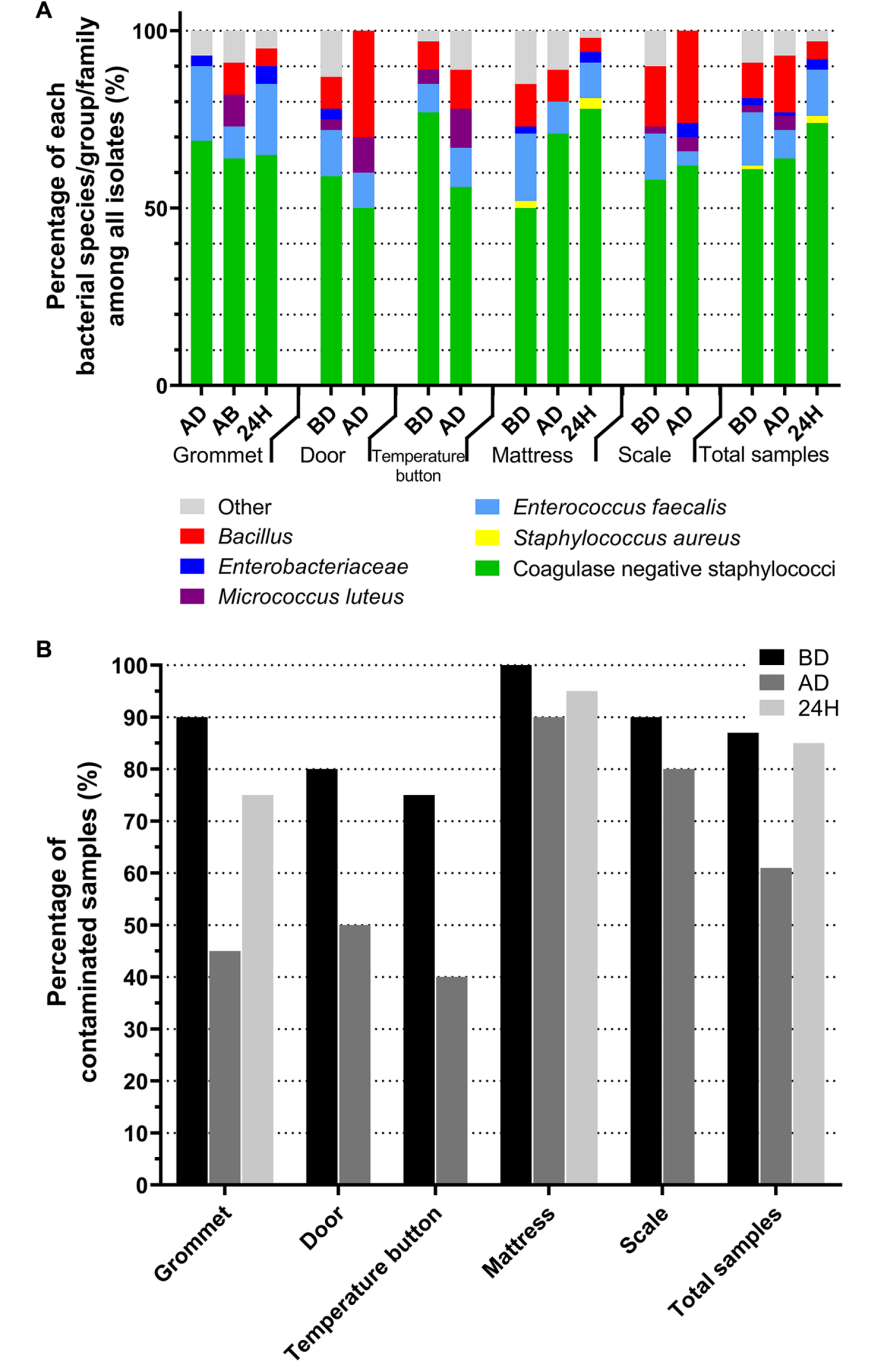
Contamination of the different incubator sites depending on the time of sampling. Each site was sampled before disinfection (BD) and after disinfection (AD). The grommet and the mattress were also sampled after 24 h during which the incubator was turned on with a neonate housed inside. **(A)** Data are presented as proportion of each bacterial species/group/family at the different times of the study, on each site. **(B)** Data are presented as the percentage of contaminated samples for each site at the different times of the study and overall

CoNS were the most frequently isolated pathogens whatever the location, with 95% contaminated incubators before disinfection, 90% after disinfection and 100% after 24 h of housing a new neonate (Figs. 1 and 2A). Among the prevalent contaminating species of CoNS before disinfection, *Staphylococcus haemolyticus* was identified in 80% of incubators followed by *Staphylococcus epidermidis* in 75% of incubators and *S. capitis* in 70% of incubators. *S. aureus* was isolated from one incubator before disinfection and was eradicated after disinfection. *Enterococcus faecalis* and *Bacillus* were isolated before disinfection from respectively 60% and 65% of incubators and after disinfection from respectively 20% and 45% of disinfected incubators. *Enterobacteriaceae* (either *Enterobacter cloacae* or *Klebsiella pneumonia* or *Escherichia coli*) were identified in 15% of incubators before disinfection and the steam pulverization conducted to their eradication except for one strain of *Klebsiella pneumoniae* that was identified on the mattress of one incubator over 20 after disinfection. Overall, the contaminated samples decreased from 87% before disinfection to 61% after disinfection (Fig. 2B).

Before disinfection a median of 4 (interquartile range [4;5]) sites per incubator among the 5 sampled sites were contaminated in the 20 incubators and this number statistically decreased after steam pulverization with a median of 3 [2;4] contaminated sites per incubator (p-value < 0.001, Wilcoxon rank test). Among the different incubator parts, the mattress and the scale were the sites that remained the most frequently contaminated after disinfection, with respectively 90% (compared to 100% before disinfection) and 80% (compared to 90% before disinfection) of positive samples (Fig. 2B). In contrast, the grommet, the temperature button and the door were more effectively disinfected as the percentage of contaminated samples decreased respectively from 90 to 45%, from 75 to 40% and from 80 to 50%.

### LOS occurrence during the study period

Neonates housed in the 20 incubators were preterm (median gestational age of 26 weeks, extremes [24–37 weeks]) with a median birth weight of 710 g [500–3210 g]. Seven (37%) were small for gestational age.

Three extreme preterm neonates with very low birth weight developed a LOS during their housing in three disinfected incubators. However, the bacterial species involved in these three LOS has not been identified from the corresponding incubator just after the disinfection process before their use. The first patient was born at 26 weeks of gestation with a birth weight of 680 g and died after three days because of a bacteremia involving *Enterobacter cloacae*. This bacterium had been isolated after 24 h of housing this patient, both from the rubber grommet and the mattress. The second patient was born at 25 weeks of gestation with a birth weight of 710 g and died after 8 days because of a pneumonia involving *Klebsiella pneumonia*. This bacterium had not been isolated from his incubator after 24 h of housing this patient. The third patient was born at 25 weeks of gestation with a birth weight of 670 g. He developed a bacteremia involving *Staphylococcus haemolyticus* after 8 days of life and he recovered. This bacterium had been isolated on the mattress after 24 h of housing this patient.

## Discussion

In the present study, we observed that steam pulverization significantly decreased the number of contaminated sites in 20 incubators of a NICU setting. However numerous sites inside incubators remained contaminated, mainly the mattresses and the scales, exposing preterm neonates to a risk of exposition to the screened pathogens.

This study was conducted after a previous one showing insufficient efficacy of another process of disinfection based on a chemical method [12]. Because other authors have reported a high efficacy of steam pulverization on both the bacterial load and the number of infections in NICUs [18–20], we hypothesized that we could be able to highlight a decreased proportion of incubator contamination in a tertiary NICU in comparison with the standard chemical protocol formerly used in this setting. The present results did not confirm this hypothesis.

However, several observations are interesting to point out. First, similarly to previous studies, the mattress remains the site that was the most difficult to disinfect [9, 12]. This is likely due to its structure since mattress presents hard-to-reach seams and closures. In another study, Cadot et al. reported that the steam could not be recommended for mattress disinfection because of the possible formation of moistures on the inner layers [13]. So, nor detergent nor steam are efficient for mattress sanitizing. This is worrying since mattress is in direct contact with neonates and can constitute a primary reservoir for pathogen involved in LOS. It seems urgent to imagine new materials and structural design for mattresses to facilitate cleaning and eradication of pathogens from its surface and seams. In that context, a recent study described the design of a starch-based polyurethane/CuO nanocomposite foam with an efficient antibacterial activity that could be used in hospital mattresses to control bacterial contamination [24]. Other solutions could result from a change in the conception of the mattress structure avoiding seams and using a waterproof material to make disinfection easier, or to use sterile single-use covers to protect the mattress from contamination. The use of low-fouling coatings inhibiting bacterial adhesion or coatings incorporating antibacterial compounds safe for neonates could also find an interesting place in the design of incubators surfaces [25]. This will potentially limit the extended contamination inside incubator and so the risk of cross contamination and nosocomial infection for neonates.

Second, we observed that, before the disinfection process, the rate of contaminated incubators sites (except the mattress) was reduced in comparison with the data from the previous study conducted in the same NICU [12]. Thus, in the present study, 87% of all the samples were contaminated before disinfection and this proportion decreased to 61% after steam disinfection, whereas in the previous study the total contamination was 96% of samples before disinfection and 69% after chemical disinfection [12]. Even if the incubators remained overall contaminated, this tendency is encouraging because it suggests that the use of steam disinfection in a NICU could progressively reduce the “chronic” bacterial colonization of incubators, probably by being more efficient on biofilms and little recesses [26]. The moderate effect reported here can be the consequence of a long period of insufficient disinfection with the previous chemical protocol [15, 21] since the incubators of the present study were the same as those of the first study and have been used in the NICU for more than 10 years. Indeed, chemical disinfection is known to damage surfaces that become porous and more difficult to clean [27]. This may have impacted our results. In the NICU where the study was performed, all incubators have been changed and replaced by new ones after the present study. It is expected that the use of disinfection with steam from the start of the use of these new incubators could show more efficacy, but further analyses are needed to confirm this hypothesis.

However, it is clear that steam pulverization did not eradicate all pathogens so disinfection process needs to be improved. A crucial element observed is that the protocol of steam pulverization requires high skills and formation, so repeated formation and audit of the caregivers in charge of incubators disinfection seems pivotal to improve its efficacy. Another specificity in the NICU of the present study is that caregivers that are in charge of disinfection are also in charge of patients. This potentially increases the risk of incubator contamination due to hand transmission but mostly decreases the expertise of the caregivers in comparison with settings where a specific personal is dedicated to the disinfection process. This organization has to be rethought to decrease the risk of incubator contamination.

Concerning the bacteria retrieved from incubators, as expected CoNS were the most frequent to colonize incubators and to resist disinfection. This was similar to the data observed with the chemical method [12]. The presence of numerous bacterial species including significant pathogens like Gram-negative bacteria after 24 h of housing a new neonate confirms that incubators are reservoirs and sources of pathogenic bacteria. It seems important to be able to clean incubators even in the presence of the neonate to decrease the bacterial load around him and thus to decrease the risk of infection. Indeed, when infected or colonized by pathogenic bacteria, neonates become the source of contamination of their own environment. In our study, three neonates developed LOS during their housing inside the incubator and two of these LOS involved a species that has been identified on the incubator after 24 h of running. We cannot exclude that these bacteria were present in another site of the incubator that has not been sampled and grow up with the moistening and heat of the incubator. This hypothesis is in agreement with the work of Hernandez-Alonso et al. who found that the recovery of *Enterobacter* from incubators after disinfection was facilitated after the incubator was running on, probably because in this condition temperature and humidity are optimal for bacterial regrowth [28]. Furthermore, the early contamination of incubators can come from caregivers and parents. So, if a better disinfection of incubators is pivotal, a respect of standard hand hygiene by all people touching newborns and environmental NICU surfaces remains central in the fight against infection of neonates [29, 30].

This work presents several limitations. First of all, only five sites were sampled for each incubator which is less than other studies and our method of analysis only provides qualitative data. These sites were chosen to be identical to the ones of the previous study, in order to be able to compare the data of these two studies [12]. The second limitation was that we did not evaluate the good handling of the steam sprayer so we cannot detect a misuse of the equipment. The third limitation was that the incubators of this study were older than 10 years so they were damaged by years of chemical exposition and routine use. It would be interesting to evaluate if steam disinfection of new incubators could show more efficacy.

## Conclusion

Even if previous studies have reported the advantages of steam pulverization in comparison with chemical methods for incubator disinfection, the present study did not show a better antimicrobial efficacy. However, the present results highlight the urgent need to develop an effective disinfection method, especially for mattresses that represent a primary reservoir for pathogen involved in LOS. Thus there is a need for substantial efforts and consultation of manufacturers to rethink incubator design and mattress conception, and to reevaluate the composition of materials used.

## Data Availability

of data and materials. The datasets used and analyzed during the current study are available from the corresponding author on reasonable request.

## List of abbreviations

NICU: Neonatal intensive care unit
LOS: Late onset sepsis
CoNS: Coagulase negative staphylococci
